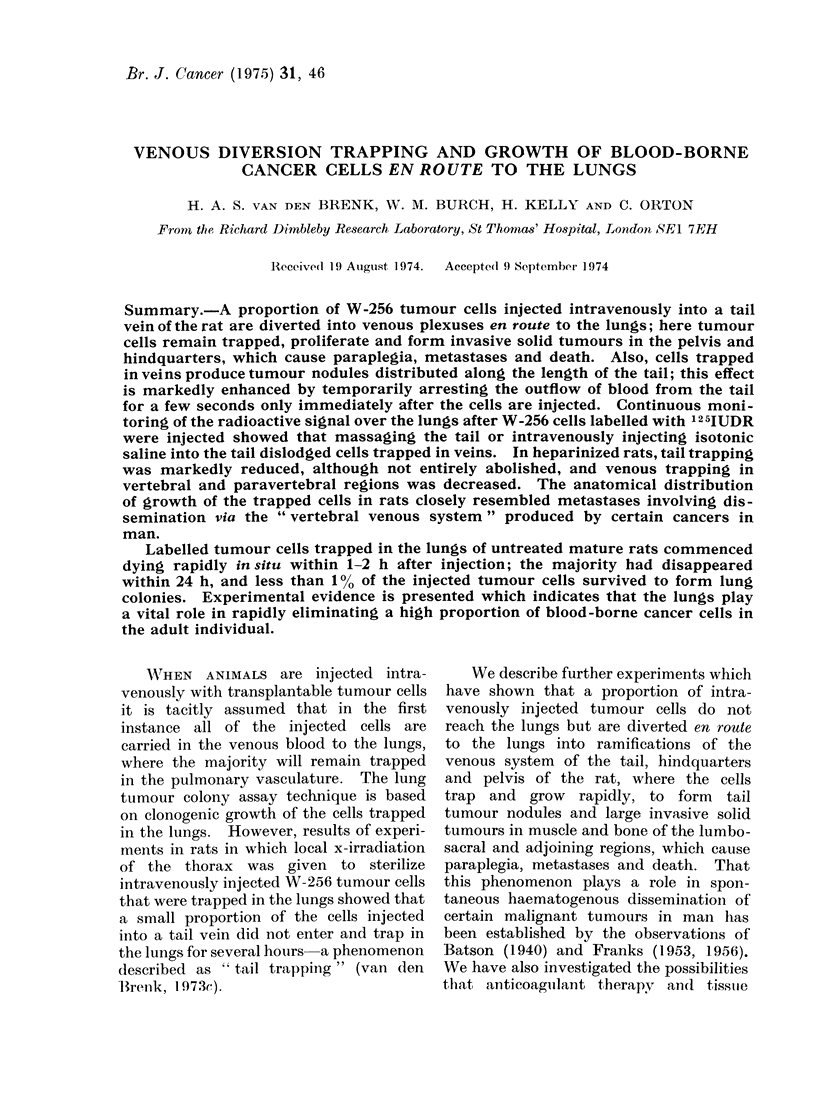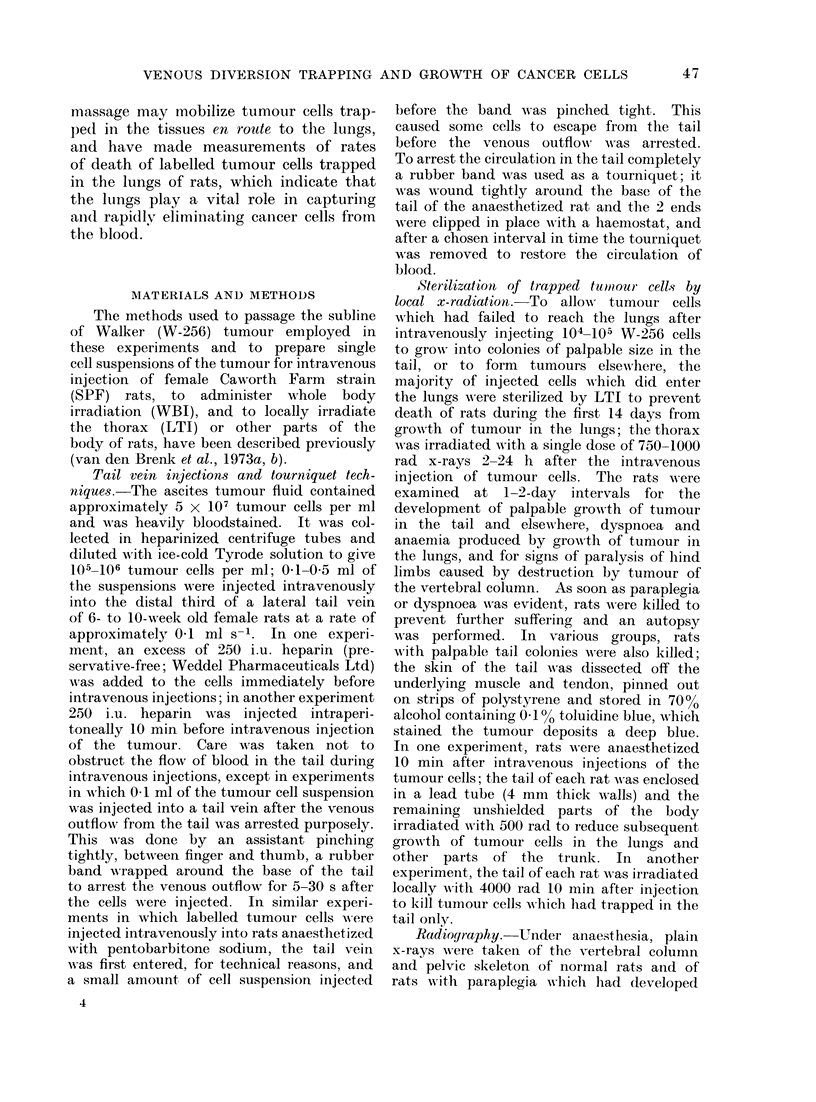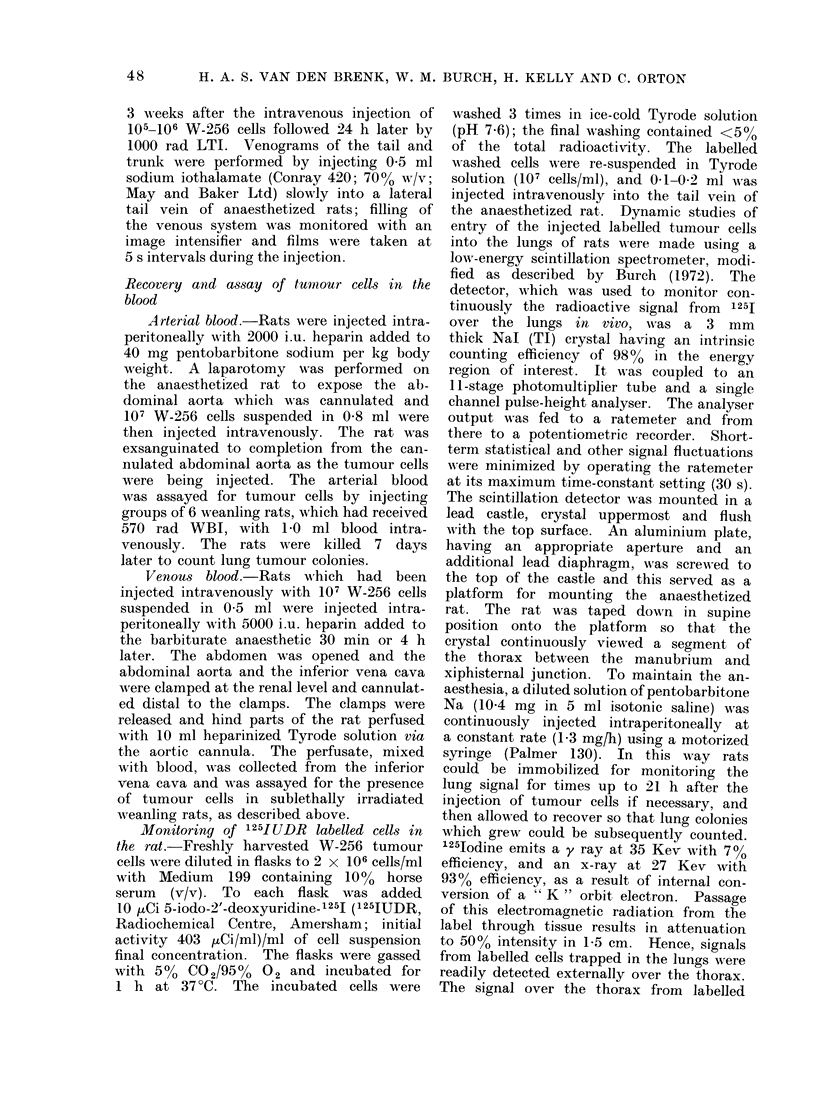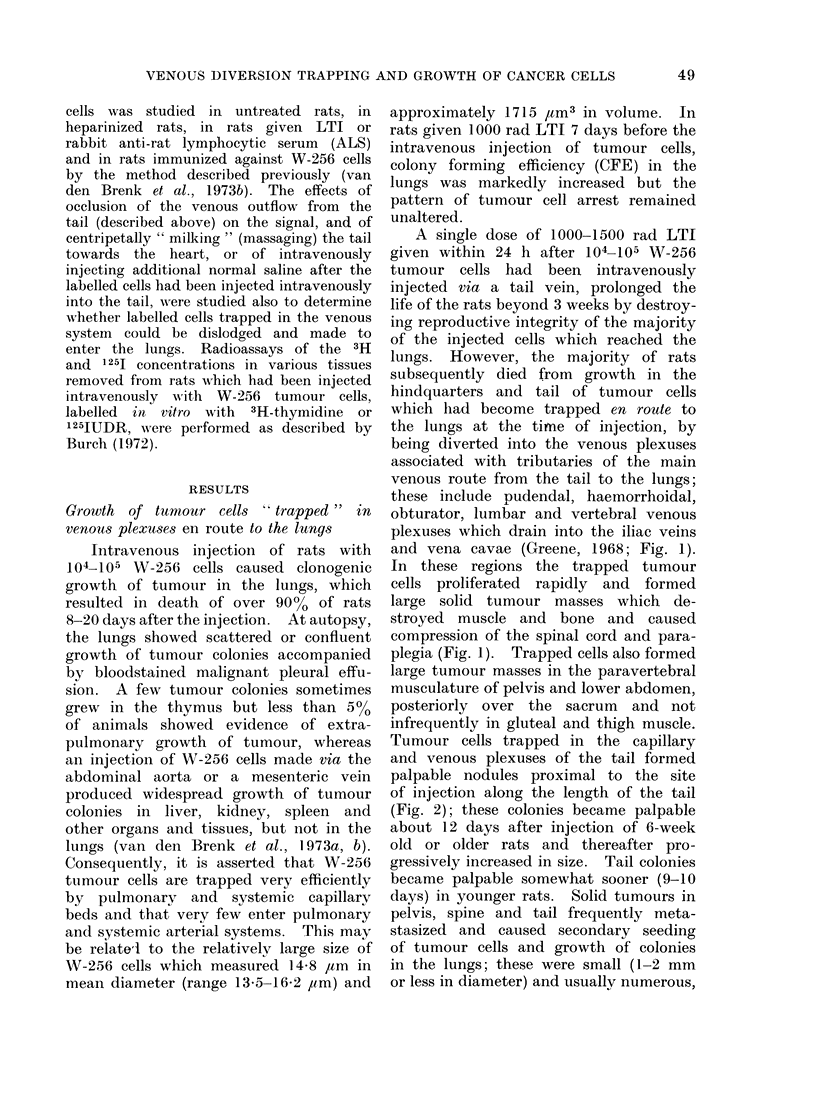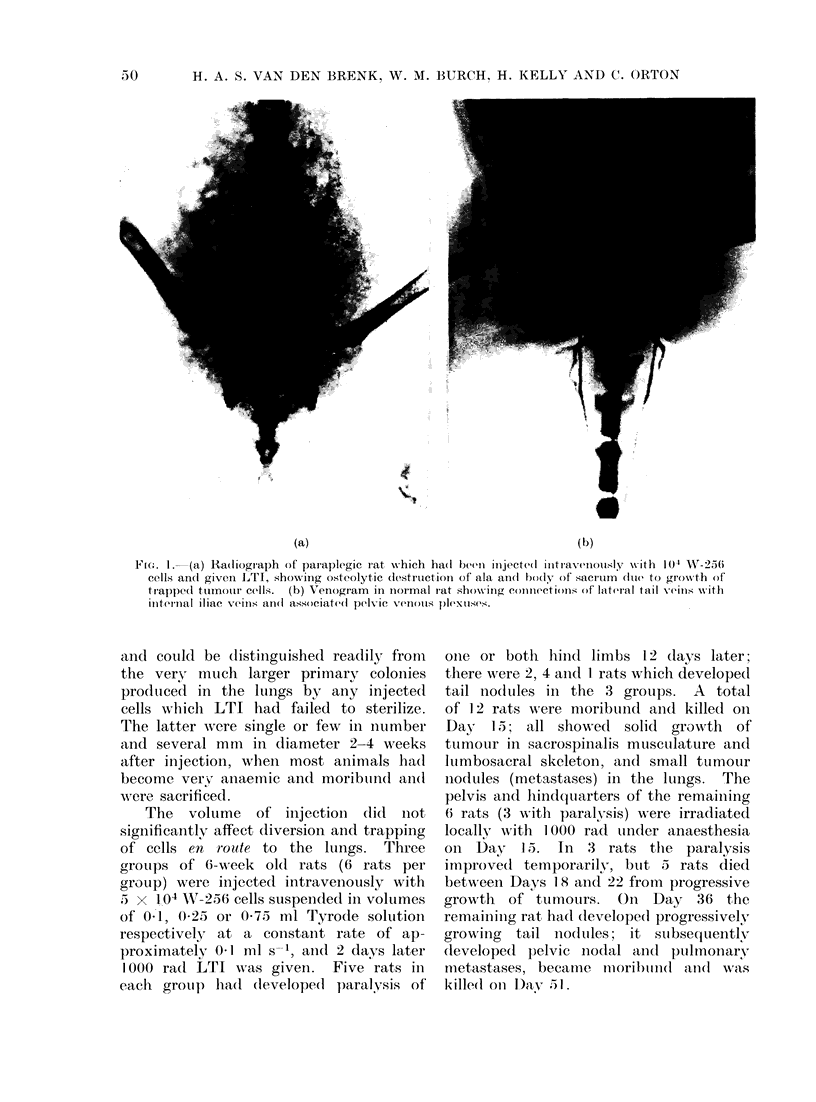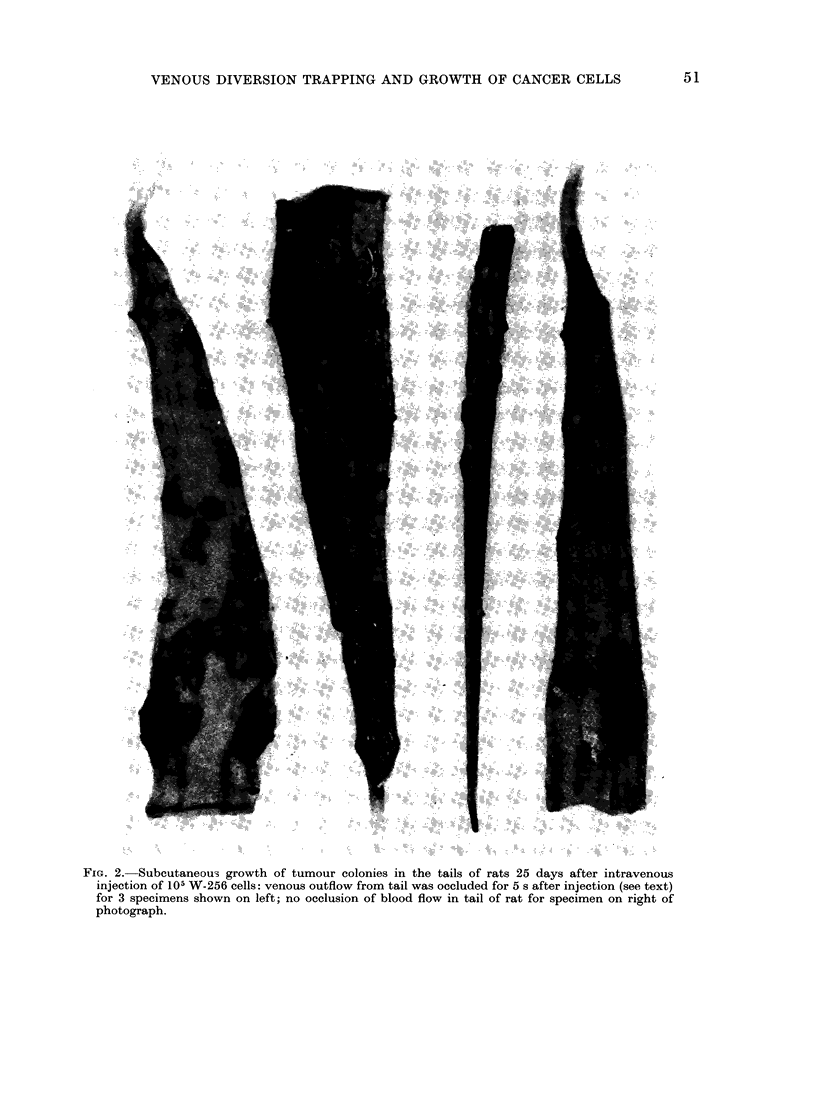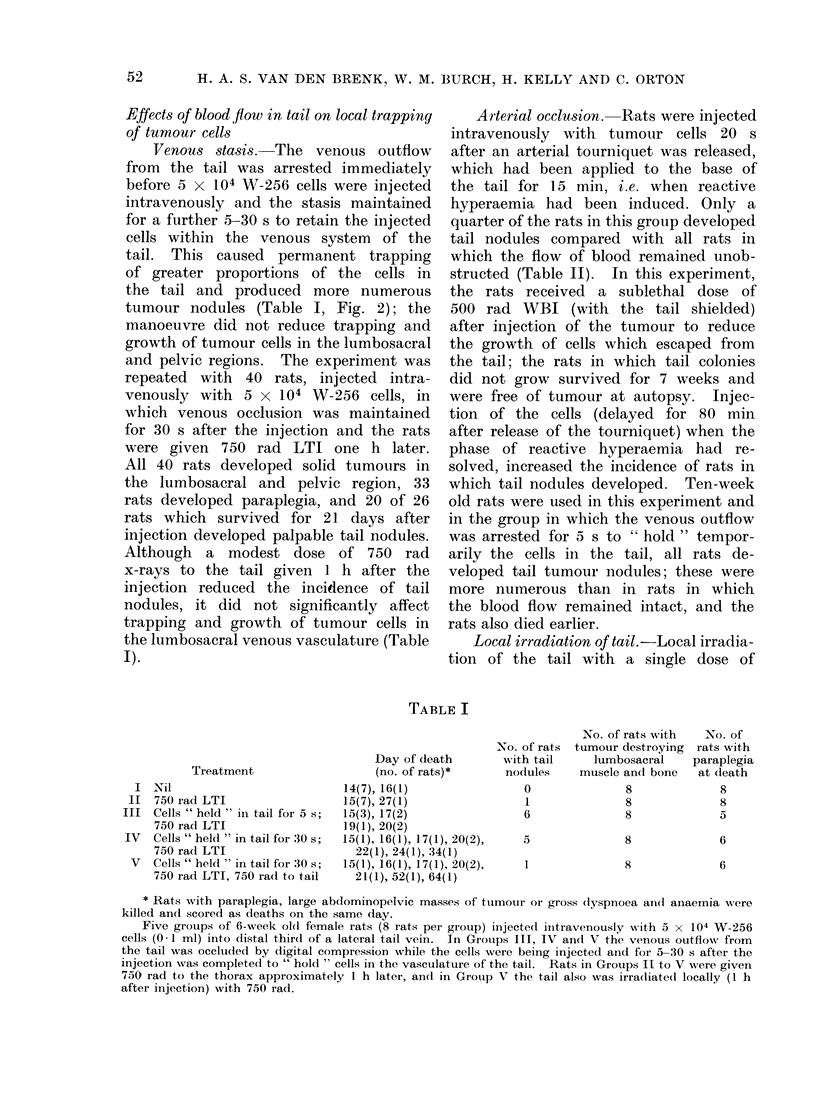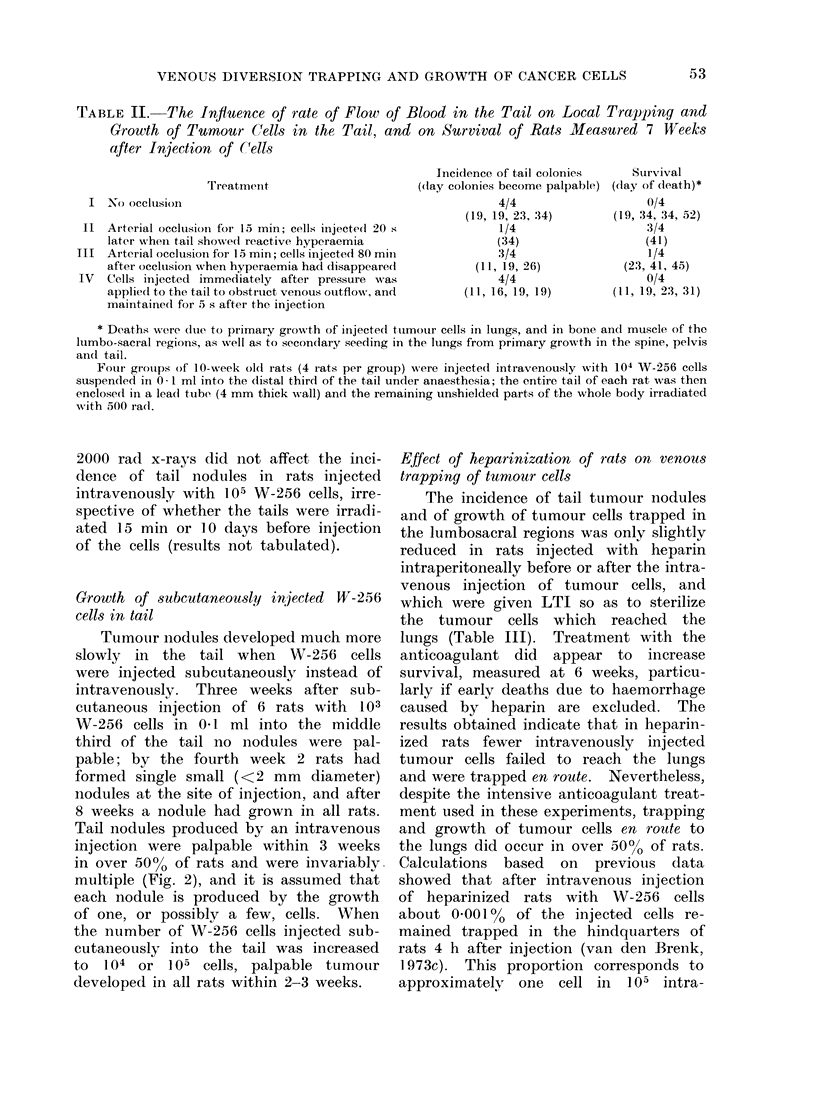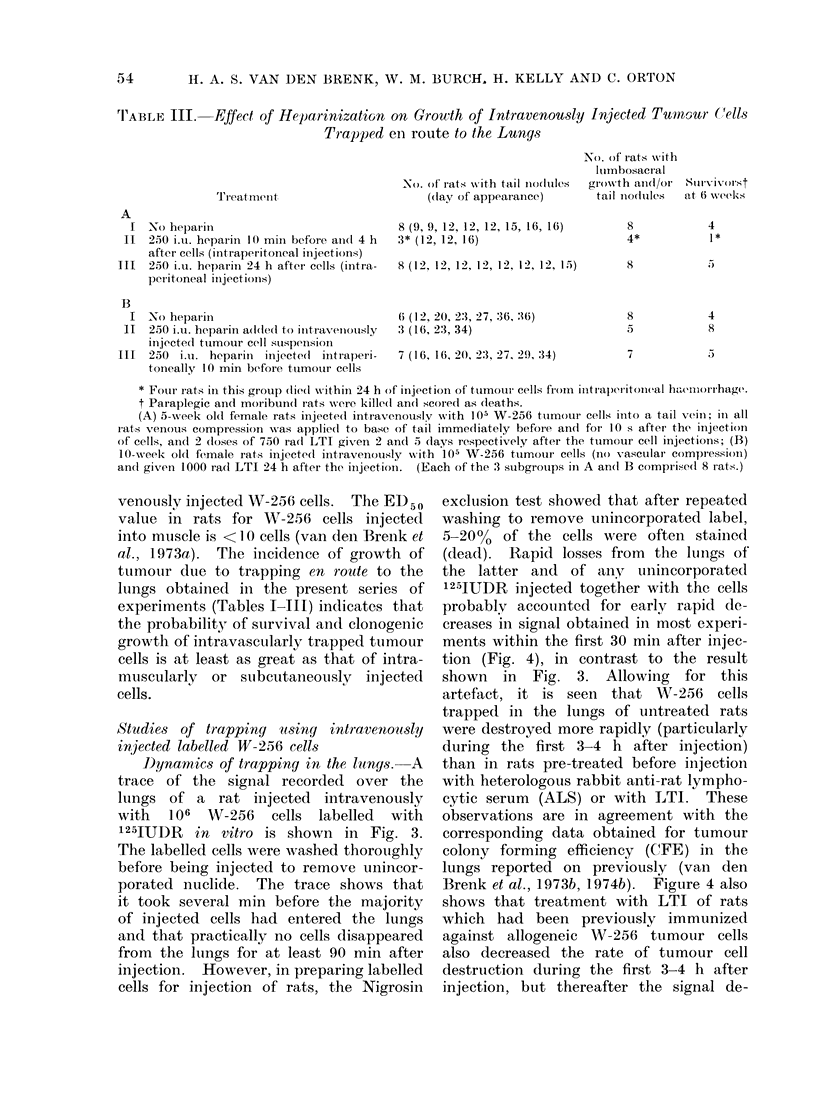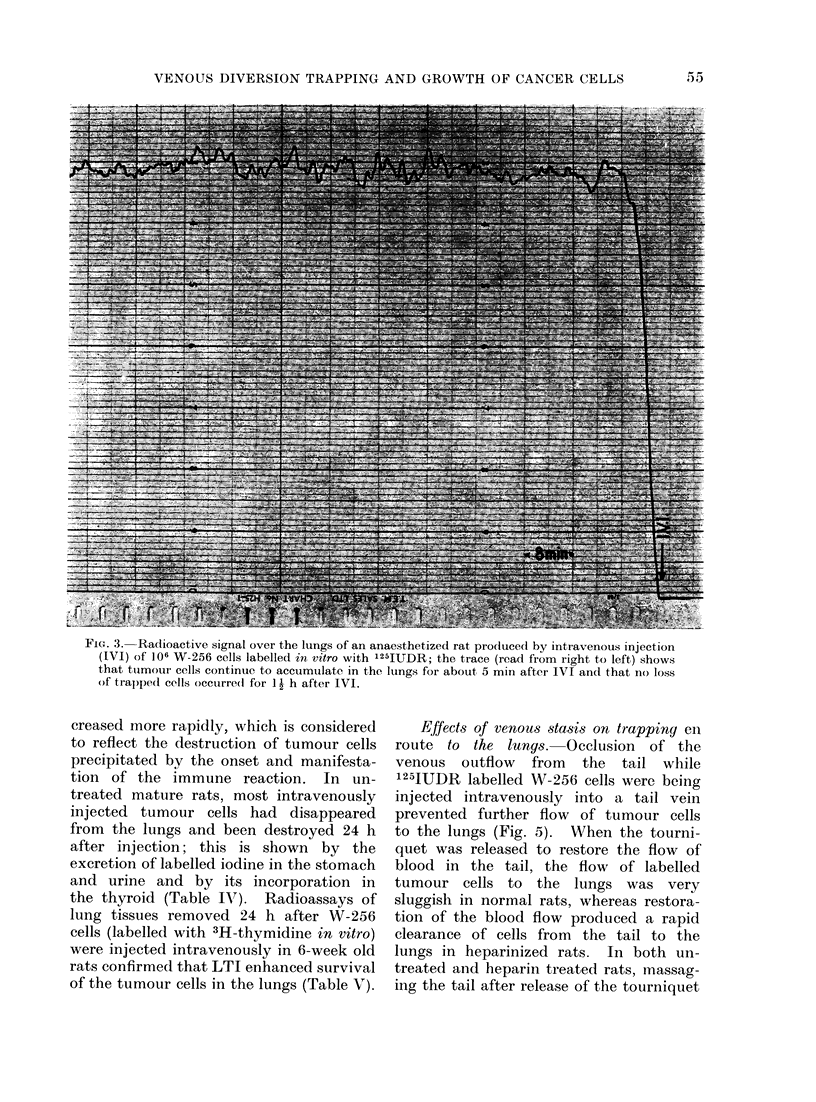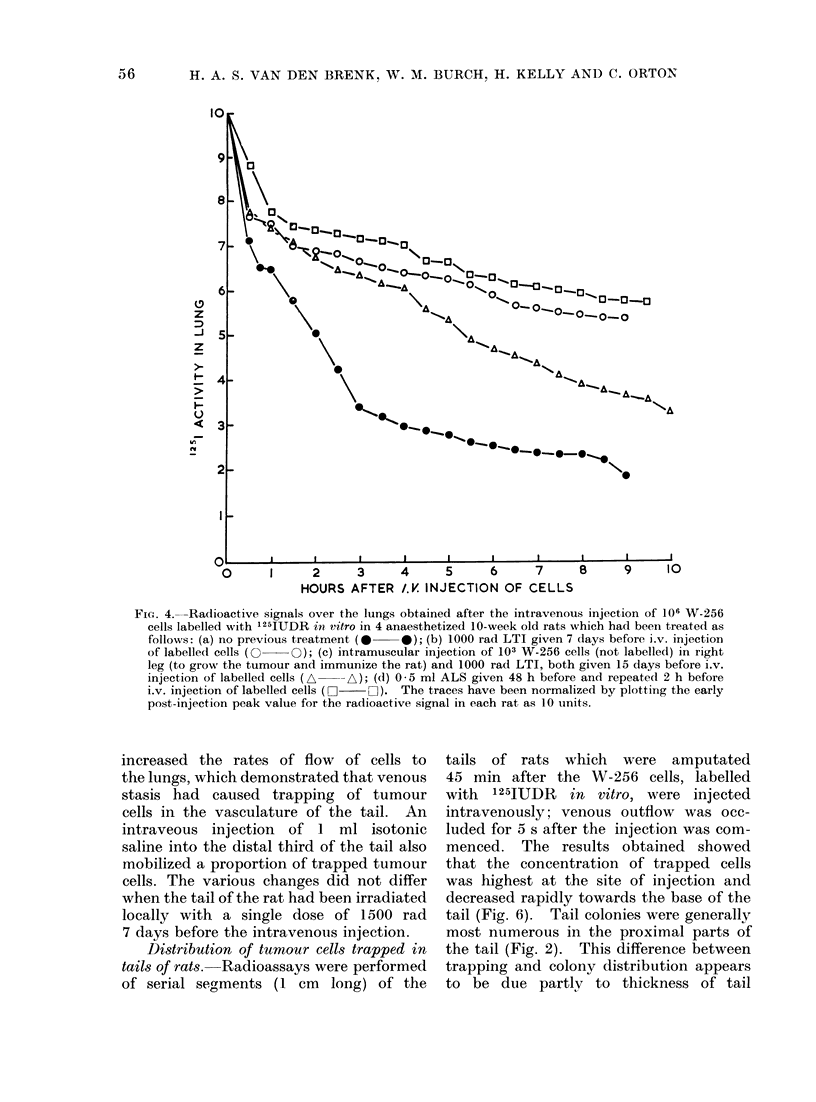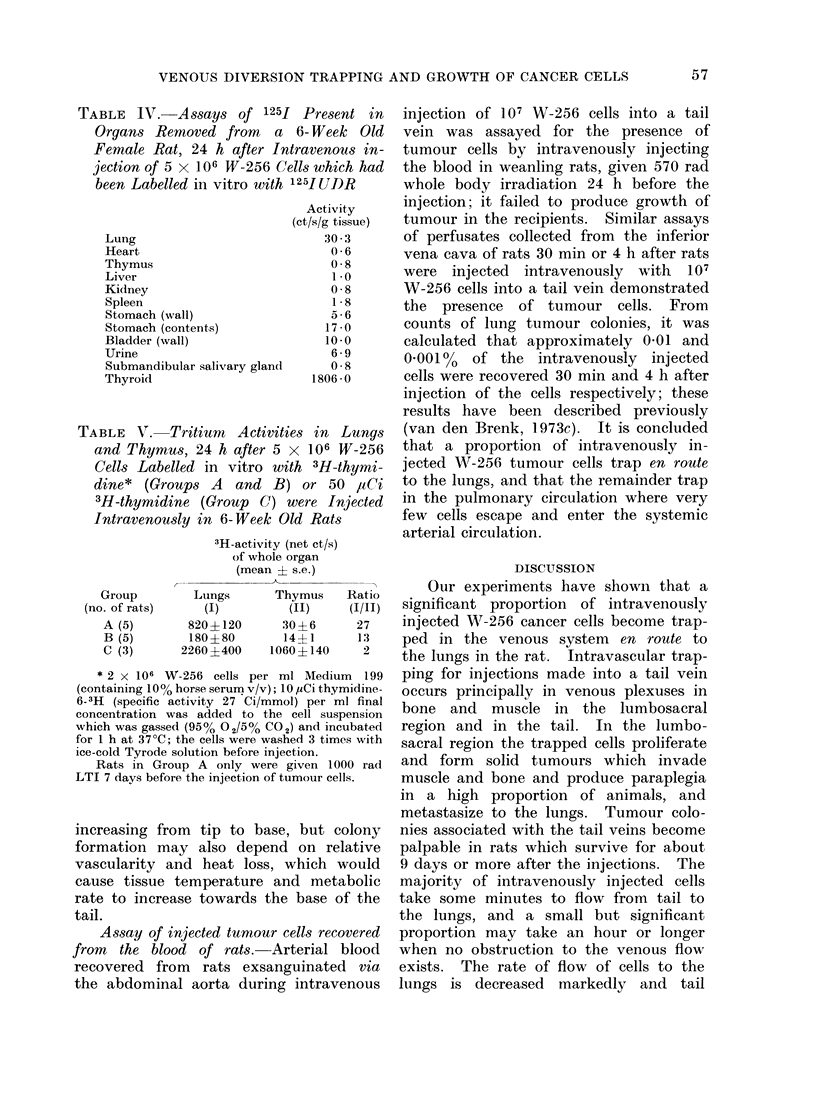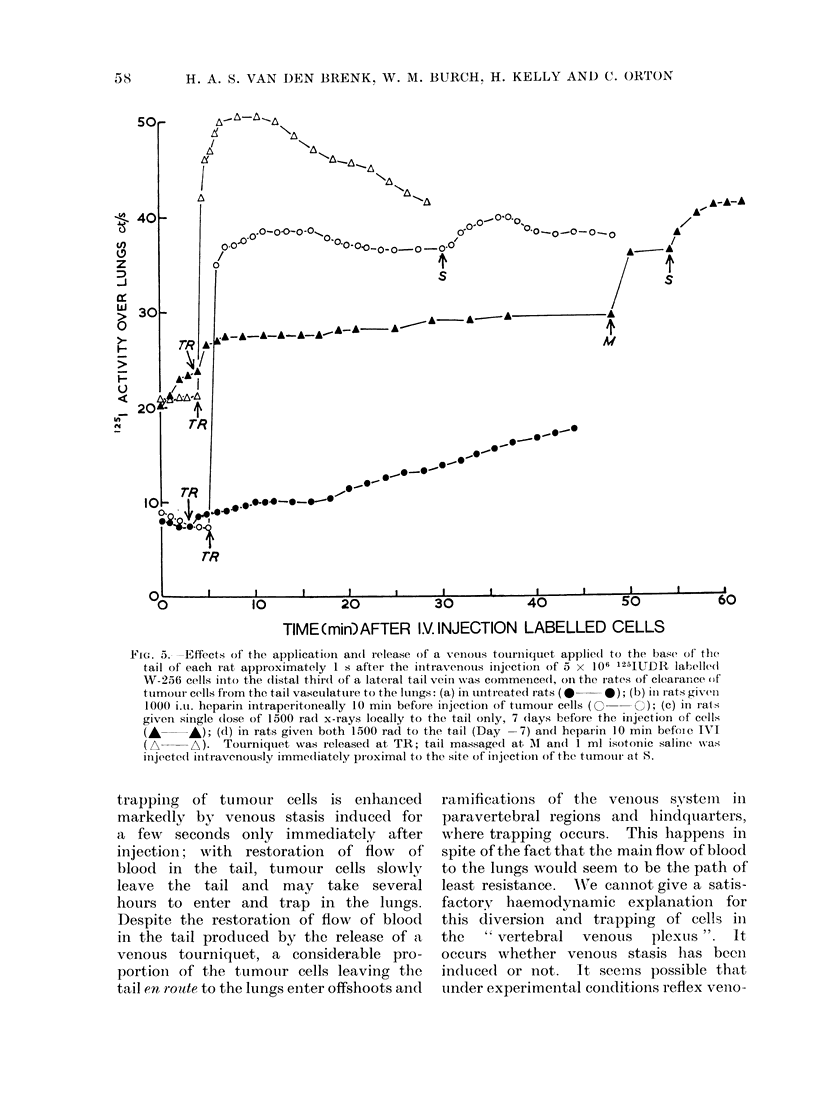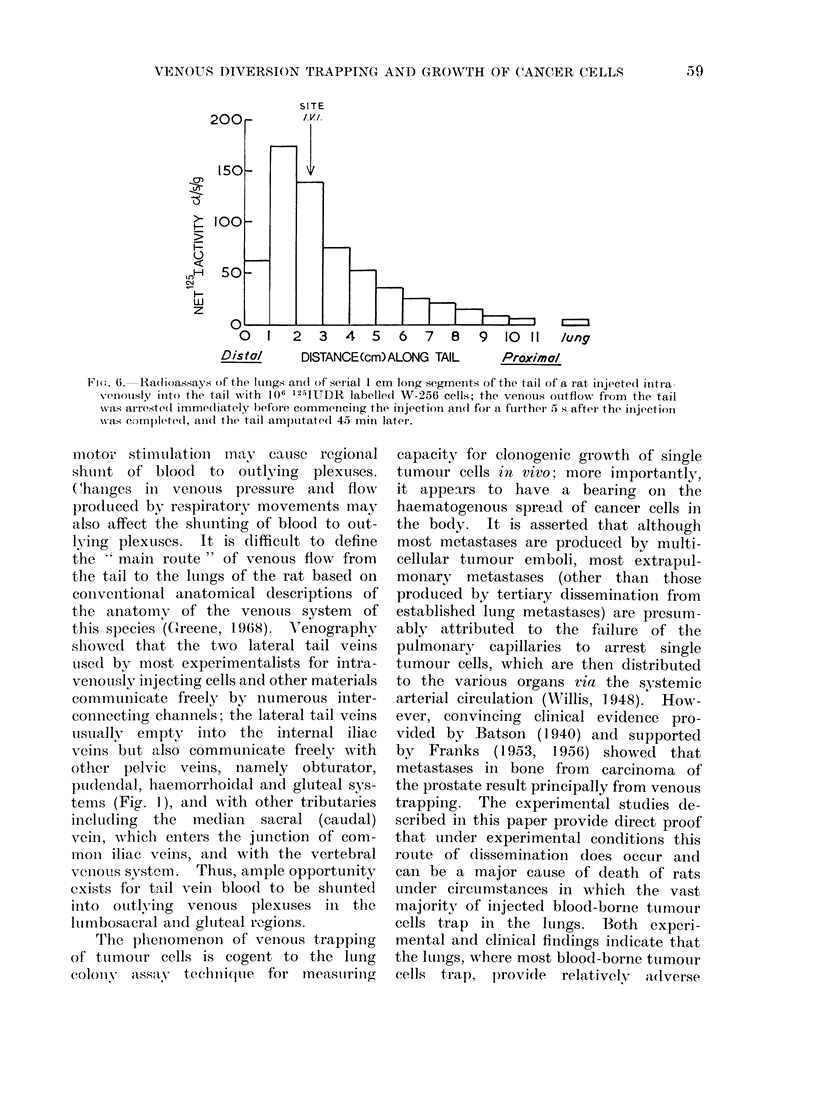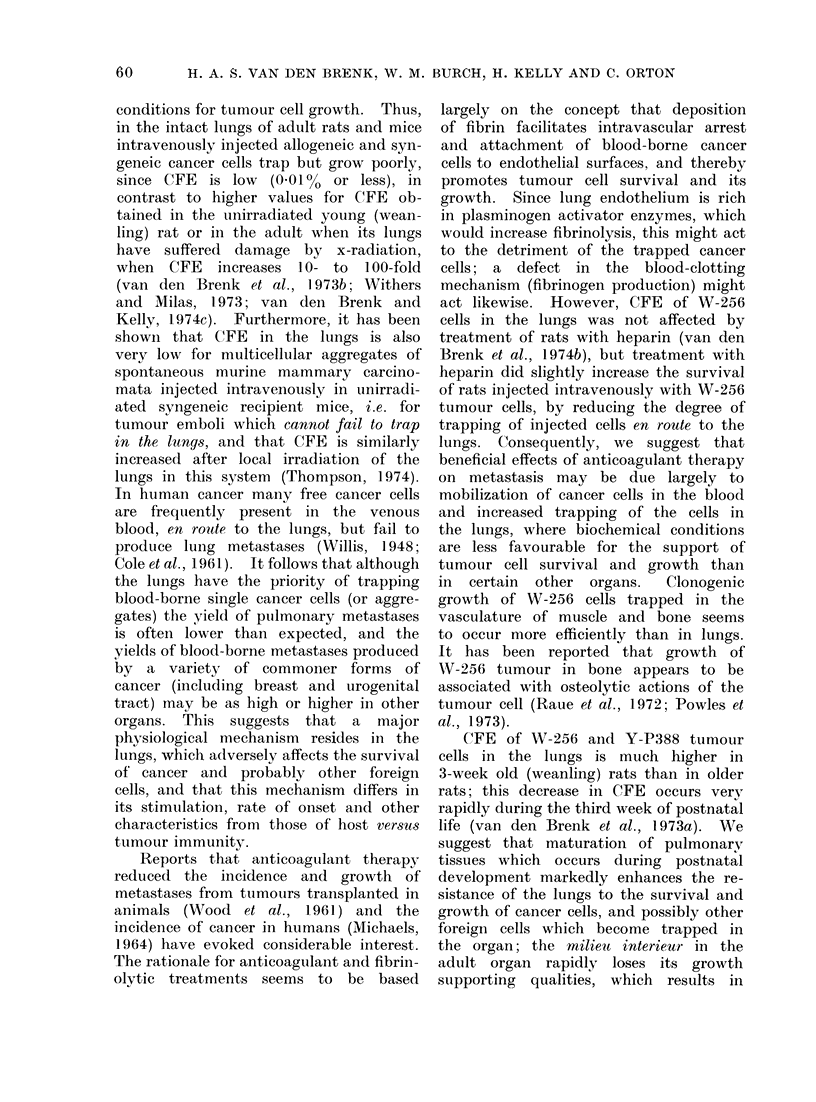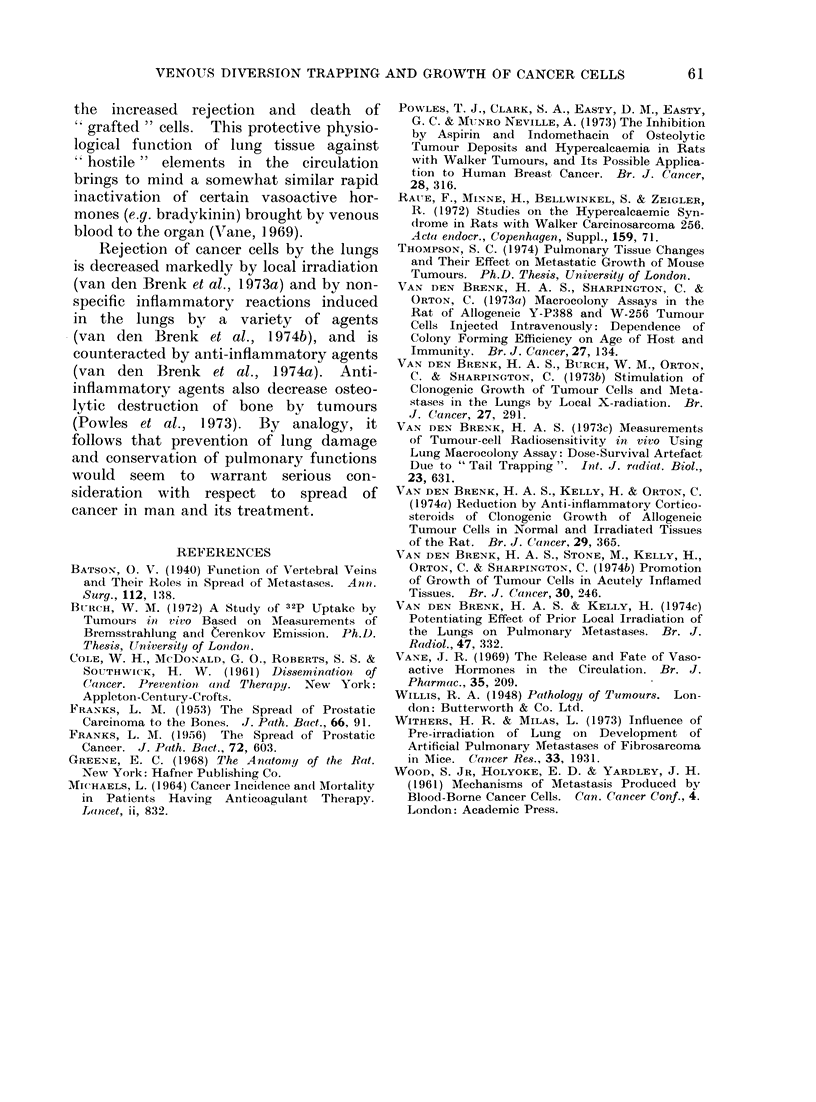# Venous diversion trapping and growth of blood-borne cancer cells en route to the lungs.

**DOI:** 10.1038/bjc.1975.6

**Published:** 1975-01

**Authors:** H. A. Van Den Brenk, W. M. Burch, H. Kelly, C. Orton

## Abstract

**Images:**


*Br. J. Cancer* (1975) **31**, 46

### VENOUS DIVERSION TRAPPING AND GROWTH OF BLOOD-BORNE CANCER CELLS *EN ROUTE* TO THE LUNGS

H. A. S. VAN DEN BRENK, W. M. BURCH, H. KELLY AND C. ORTON

*From the Richard Dimbleby Research Laboratory, St Thomas' Hospital, London SE1 7EH*

Received 19 August 1974. Accepted 9 September 1974

**Summary.—A proportion of W-256 tumour cells injected intravenously into a tail vein of the rat are diverted into venous plexuses *en route* to the lungs; here tumour cells remain trapped, proliferate and form invasive solid tumours in the pelvis and hindquarters, which cause paraplegia, metastases and death. Also, cells trapped in veins produce tumour nodules distributed along the length of the tail; this effect is markedly enhanced by temporarily arresting the outflow of blood from the tail for a few seconds only immediately after the cells are injected. Continuous monitoring of the radioactive signal over the lungs after W-256 cells labelled with $^{125}$IUDR were injected showed that massaging the tail or intravenously injecting isotonic saline into the tail dislodged cells trapped in veins. In heparinized rats, tail trapping was markedly reduced, although not entirely abolished, and venous trapping in vertebral and paravertebral regions was decreased. The anatomical distribution of growth of the trapped cells in rats closely resembled metastases involving dissemination *via* the "vertebral venous system" produced by certain cancers in man.**

**Labelled tumour cells trapped in the lungs of untreated mature rats commenced dying rapidly *in situ* within 1–2 h after injection; the majority had disappeared within 24 h, and less than 1% of the injected tumour cells survived to form lung colonies. Experimental evidence is presented which indicates that the lungs play a vital role in rapidly eliminating a high proportion of blood-borne cancer cells in the adult individual.**

WHEN ANIMALS are injected intravenously with transplantable tumour cells it is tacitly assumed that in the first instance all of the injected cells are carried in the venous blood to the lungs, where the majority will remain trapped in the pulmonary vasculature. The lung tumour colony assay technique is based on clonogenic growth of the cells trapped in the lungs. However, results of experiments in rats in which local x-irradiation of the thorax was given to sterilize intravenously injected W-256 tumour cells that were trapped in the lungs showed that a small proportion of the cells injected into a tail vein did not enter and trap in the lungs for several hours—a phenomenon described as "tail trapping" (van den Brenk, 1973*c*).

We describe further experiments which have shown that a proportion of intravenously injected tumour cells do not reach the lungs but are diverted *en route* to the lungs into ramifications of the venous system of the tail, hindquarters and pelvis of the rat, where the cells trap and grow rapidly, to form tail tumour nodules and large invasive solid tumours in muscle and bone of the lumbosacral and adjoining regions, which cause paraplegia, metastases and death. That this phenomenon plays a role in spontaneous haematogenous dissemination of certain malignant tumours in man has been established by the observations of Batson (1940) and Franks (1953, 1956). We have also investigated the possibilities that anticoagulant therapy and tissue massage may mobilize tumour cells trapped in the tissues *en route* to the lungs, and have made measurements of rates of death of labelled tumour cells trapped in the lungs of rats, which indicate that the lungs play a vital role in capturing and rapidly eliminating cancer cells from the blood.

### MATERIALS AND METHODS

The methods used to passage the subline of Walker (W-256) tumour employed in these experiments and to prepare single cell suspensions of the tumour for intravenous injection of female Caworth Farm strain (SPF) rats, to administer whole body irradiation (WBI), and to locally irradiate the thorax (LTI) or other parts of the body of rats, have been described previously (van den Brenk *et al.*, 1973*a*, *b*).

*Tail vein injections and tourniquet techniques.*—The ascites tumour fluid contained approximately $5 \times 10^7$ tumour cells per ml and was heavily bloodstained. It was collected in heparinized centrifuge tubes and diluted with ice-cold Tyrode solution to give $10^5$–$10^6$ tumour cells per ml; 0·1–0·5 ml of the suspensions were injected intravenously into the distal third of a lateral tail vein of 6- to 10-week old female rats at a rate of approximately 0·1 ml $s^{-1}$. In one experiment, an excess of 250 i.u. heparin (preservative-free; Weddel Pharmaceuticals Ltd) was added to the cells immediately before intravenous injections; in another experiment 250 i.u. heparin was injected intraperitoneally 10 min before intravenous injection of the tumour. Care was taken not to obstruct the flow of blood in the tail during intravenous injections, except in experiments in which 0·1 ml of the tumour cell suspension was injected into a tail vein after the venous outflow from the tail was arrested purposely. This was done by an assistant pinching tightly, between finger and thumb, a rubber band wrapped around the base of the tail to arrest the venous outflow for 5–30 s after the cells were injected. In similar experiments in which labelled tumour cells were injected intravenously into rats anaesthetized with pentobarbitone sodium, the tail vein was first entered, for technical reasons, and a small amount of cell suspension injected before the band was pinched tight. This caused some cells to escape from the tail before the venous outflow was arrested. To arrest the circulation in the tail completely a rubber band was used as a tourniquet; it was wound tightly around the base of the tail of the anaesthetized rat and the 2 ends were clipped in place with a haemostat, and after a chosen interval in time the tourniquet was removed to restore the circulation of blood.

*Sterilization of trapped tumour cells by local x-radiation.*—To allow tumour cells which had failed to reach the lungs after intravenously injecting $10^4$–$10^5$ W-256 cells to grow into colonies of palpable size in the tail, or to form tumours elsewhere, the majority of injected cells which did enter the lungs were sterilized by LTI to prevent death of rats during the first 14 days from growth of tumour in the lungs; the thorax was irradiated with a single dose of 750–1000 rad x-rays 2–24 h after the intravenous injection of tumour cells. The rats were examined at 1–2-day intervals for the development of palpable growth of tumour in the tail and elsewhere, dyspnoea and anaemia produced by growth of tumour in the lungs, and for signs of paralysis of hind limbs caused by destruction by tumour of the vertebral column. As soon as paraplegia or dyspnoea was evident, rats were killed to prevent further suffering and an autopsy was performed. In various groups, rats with palpable tail colonies were also killed; the skin of the tail was dissected off the underlying muscle and tendon, pinned out on strips of polystyrene and stored in 70% alcohol containing 0·1% toluidine blue, which stained the tumour deposits a deep blue. In one experiment, rats were anaesthetized 10 min after intravenous injections of the tumour cells; the tail of each rat was enclosed in a lead tube (4 mm thick walls) and the remaining unshielded parts of the body irradiated with 500 rad to reduce subsequent growth of tumour cells in the lungs and other parts of the trunk. In another experiment, the tail of each rat was irradiated locally with 4000 rad 10 min after injection to kill tumour cells which had trapped in the tail only.

*Radiography.*—Under anaesthesia, plain x-rays were taken of the vertebral column and pelvic skeleton of normal rats and of rats with paraplegia which had developed

3 weeks after the intravenous injection of $10^5$–$10^6$ W-256 cells followed 24 h later by 1000 rad LTI. Venograms of the tail and trunk were performed by injecting 0·5 ml sodium iothalamate (Conray 420; 70% w/v; May and Baker Ltd) slowly into a lateral tail vein of anaesthetized rats; filling of the venous system was monitored with an image intensifier and films were taken at 5 s intervals during the injection.

*Recovery and assay of tumour cells in the blood*

*Arterial blood.*—Rats were injected intraperitoneally with 2000 i.u. heparin added to 40 mg pentobarbitone sodium per kg body weight. A laparotomy was performed on the anaesthetized rat to expose the abdominal aorta which was cannulated and $10^7$ W-256 cells suspended in 0·8 ml were then injected intravenously. The rat was exsanguinated to completion from the cannulated abdominal aorta as the tumour cells were being injected. The arterial blood was assayed for tumour cells by injecting groups of 6 weanling rats, which had received 570 rad WBI, with 1·0 ml blood intravenously. The rats were killed 7 days later to count lung tumour colonies.

*Venous blood.*—Rats which had been injected intravenously with $10^7$ W-256 cells suspended in 0·5 ml were injected intraperitoneally with 5000 i.u. heparin added to the barbiturate anaesthetic 30 min or 4 h later. The abdomen was opened and the abdominal aorta and the inferior vena cava were clamped at the renal level and cannulated distal to the clamps. The clamps were released and hind parts of the rat perfused with 10 ml heparinized Tyrode solution *via* the aortic cannula. The perfusate, mixed with blood, was collected from the inferior vena cava and was assayed for the presence of tumour cells in sublethally irradiated weanling rats, as described above.

*Monitoring of $^{125}$IUDR labelled cells in the rat.*—Freshly harvested W-256 tumour cells were diluted in flasks to $2 \times 10^6$ cells/ml with Medium 199 containing 10% horse serum (v/v). To each flask was added 10 $\mu$Ci 5-iodo-2′-deoxyuridine-$^{125}$I ($^{125}$IUDR, Radiochemical Centre, Amersham; initial activity 403 $\mu$Ci/ml)/ml of cell suspension final concentration. The flasks were gassed with 5% $CO_2$/95% $O_2$ and incubated for 1 h at 37°C. The incubated cells were washed 3 times in ice-cold Tyrode solution (pH 7·6); the final washing contained <5% of the total radioactivity. The labelled washed cells were re-suspended in Tyrode solution ($10^7$ cells/ml), and 0·1–0·2 ml was injected intravenously into the tail vein of the anaesthetized rat. Dynamic studies of entry of the injected labelled tumour cells into the lungs of rats were made using a low-energy scintillation spectrometer, modified as described by Burch (1972). The detector, which was used to monitor continuously the radioactive signal from $^{125}$I over the lungs *in vivo*, was a 3 mm thick NaI (TI) crystal having an intrinsic counting efficiency of 98% in the energy region of interest. It was coupled to an 11-stage photomultiplier tube and a single channel pulse-height analyser. The analyser output was fed to a ratemeter and from there to a potentiometric recorder. Short-term statistical and other signal fluctuations were minimized by operating the ratemeter at its maximum time-constant setting (30 s). The scintillation detector was mounted in a lead castle, crystal uppermost and flush with the top surface. An aluminium plate, having an appropriate aperture and an additional lead diaphragm, was screwed to the top of the castle and this served as a platform for mounting the anaesthetized rat. The rat was taped down in supine position onto the platform so that the crystal continuously viewed a segment of the thorax between the manubrium and xiphisternal junction. To maintain the anaesthesia, a diluted solution of pentobarbitone Na (10·4 mg in 5 ml isotonic saline) was continuously injected intraperitoneally at a constant rate (1·3 mg/h) using a motorized syringe (Palmer 130). In this way rats could be immobilized for monitoring the lung signal for times up to 21 h after the injection of tumour cells if necessary, and then allowed to recover so that lung colonies which grew could be subsequently counted. $^{125}$Iodine emits a $\gamma$ ray at 35 Kev with 7% efficiency, and an x-ray at 27 Kev with 93% efficiency, as a result of internal conversion of a " K " orbit electron. Passage of this electromagnetic radiation from the label through tissue results in attenuation to 50% intensity in 1·5 cm. Hence, signals from labelled cells trapped in the lungs were readily detected externally over the thorax. The signal over the thorax from labelled cells was studied in untreated rats, in heparinized rats, in rats given LTI or rabbit anti-rat lymphocytic serum (ALS) and in rats immunized against W-256 cells by the method described previously (van den Brenk *et al.*, 1973*b*). The effects of occlusion of the venous outflow from the tail (described above) on the signal, and of centripetally " milking " (massaging) the tail towards the heart, or of intravenously injecting additional normal saline after the labelled cells had been injected intravenously into the tail, were studied also to determine whether labelled cells trapped in the venous system could be dislodged and made to enter the lungs. Radioassays of the $^{3}H$ and $^{125}I$ concentrations in various tissues removed from rats which had been injected intravenously with W-256 tumour cells, labelled *in vitro* with $^{3}H$-thymidine or $^{125}$IUDR, were performed as described by Burch (1972).

### RESULTS

### *Growth of tumour cells " trapped " in venous plexuses* en route *to the lungs*

Intravenous injection of rats with $10^{4}$–$10^{5}$ W-256 cells caused clonogenic growth of tumour in the lungs, which resulted in death of over 90% of rats 8–20 days after the injection. At autopsy, the lungs showed scattered or confluent growth of tumour colonies accompanied by bloodstained malignant pleural effusion. A few tumour colonies sometimes grew in the thymus but less than 5% of animals showed evidence of extra-pulmonary growth of tumour, whereas an injection of W-256 cells made *via* the abdominal aorta or a mesenteric vein produced widespread growth of tumour colonies in liver, kidney, spleen and other organs and tissues, but not in the lungs (van den Brenk *et al.*, 1973*a*, *b*). Consequently, it is asserted that W-256 tumour cells are trapped very efficiently by pulmonary and systemic capillary beds and that very few enter pulmonary and systemic arterial systems. This may be related to the relatively large size of W-256 cells which measured 14·8 $\mu$m in mean diameter (range 13·5–16·2 $\mu$m) and approximately 1715 $\mu m^3$ in volume. In rats given 1000 rad LTI 7 days before the intravenous injection of tumour cells, colony forming efficiency (CFE) in the lungs was markedly increased but the pattern of tumour cell arrest remained unaltered.

A single dose of 1000–1500 rad LTI given within 24 h after $10^{4}$–$10^{5}$ W-256 tumour cells had been intravenously injected *via* a tail vein, prolonged the life of the rats beyond 3 weeks by destroying reproductive integrity of the majority of the injected cells which reached the lungs. However, the majority of rats subsequently died from growth in the hindquarters and tail of tumour cells which had become trapped *en route* to the lungs at the time of injection, by being diverted into the venous plexuses associated with tributaries of the main venous route from the tail to the lungs; these include pudendal, haemorrhoidal, obturator, lumbar and vertebral venous plexuses which drain into the iliac veins and vena cavae (Greene, 1968; Fig. 1). In these regions the trapped tumour cells proliferated rapidly and formed large solid tumour masses which destroyed muscle and bone and caused compression of the spinal cord and paraplegia (Fig. 1). Trapped cells also formed large tumour masses in the paravertebral musculature of pelvis and lower abdomen, posteriorly over the sacrum and not infrequently in gluteal and thigh muscle. Tumour cells trapped in the capillary and venous plexuses of the tail formed palpable nodules proximal to the site of injection along the length of the tail (Fig. 2); these colonies became palpable about 12 days after injection of 6-week old or older rats and thereafter progressively increased in size. Tail colonies became palpable somewhat sooner (9–10 days) in younger rats. Solid tumours in pelvis, spine and tail frequently metastasized and caused secondary seeding of tumour cells and growth of colonies in the lungs; these were small (1–2 mm or less in diameter) and usually numerous,

(a) (b)

FIG. 1.—(a) Radiograph of paraplegic rat which had been injected intravenously with $10^4$ W-256 cells and given LTI, showing osteolytic destruction of ala and body of sacrum due to growth of trapped tumour cells. (b) Venogram in normal rat showing connections of lateral tail veins with internal iliac veins and associated pelvic venous plexuses.

and could be distinguished readily from the very much larger primary colonies produced in the lungs by any injected cells which LTI had failed to sterilize. The latter were single or few in number and several mm in diameter 2–4 weeks after injection, when most animals had become very anaemic and moribund and were sacrificed.

The volume of injection did not significantly affect diversion and trapping of cells *en route* to the lungs. Three groups of 6-week old rats (6 rats per group) were injected intravenously with $5 \times 10^4$ W-256 cells suspended in volumes of 0·1, 0·25 or 0·75 ml Tyrode solution respectively at a constant rate of approximately 0·1 ml $s^{-1}$, and 2 days later 1000 rad LTI was given. Five rats in each group had developed paralysis of one or both hind limbs 12 days later; there were 2, 4 and 1 rats which developed tail nodules in the 3 groups. A total of 12 rats were moribund and killed on Day 15; all showed solid growth of tumour in sacrospinalis musculature and lumbosacral skeleton, and small tumour nodules (metastases) in the lungs. The pelvis and hindquarters of the remaining 6 rats (3 with paralysis) were irradiated locally with 1000 rad under anaesthesia on Day 15. In 3 rats the paralysis improved temporarily, but 5 rats died between Days 18 and 22 from progressive growth of tumours. On Day 36 the remaining rat had developed progressively growing tail nodules; it subsequently developed pelvic nodal and pulmonary metastases, became moribund and was killed on Day 51.

FIG. 2.—Subcutaneous growth of tumour colonies in the tails of rats 25 days after intravenous injection of $10^5$ W-256 cells: venous outflow from tail was occluded for 5 s after injection (see text) for 3 specimens shown on left; no occlusion of blood flow in tail of rat for specimen on right of photograph.

*Effects of blood flow in tail on local trapping of tumour cells*

*Venous stasis.*—The venous outflow from the tail was arrested immediately before $5 \times 10^4$ W-256 cells were injected intravenously and the stasis maintained for a further 5–30 s to retain the injected cells within the venous system of the tail. This caused permanent trapping of greater proportions of the cells in the tail and produced more numerous tumour nodules (Table I, Fig. 2); the manoeuvre did not reduce trapping and growth of tumour cells in the lumbosacral and pelvic regions. The experiment was repeated with 40 rats, injected intravenously with $5 \times 10^4$ W-256 cells, in which venous occlusion was maintained for 30 s after the injection and the rats were given 750 rad LTI one h later. All 40 rats developed solid tumours in the lumbosacral and pelvic region, 33 rats developed paraplegia, and 20 of 26 rats which survived for 21 days after injection developed palpable tail nodules. Although a modest dose of 750 rad x-rays to the tail given 1 h after the injection reduced the incidence of tail nodules, it did not significantly affect trapping and growth of tumour cells in the lumbosacral venous vasculature (Table I).

*Arterial occlusion.*—Rats were injected intravenously with tumour cells 20 s after an arterial tourniquet was released, which had been applied to the base of the tail for 15 min, *i.e.* when reactive hyperaemia had been induced. Only a quarter of the rats in this group developed tail nodules compared with all rats in which the flow of blood remained unobstructed (Table II). In this experiment, the rats received a sublethal dose of 500 rad WBI (with the tail shielded) after injection of the tumour to reduce the growth of cells which escaped from the tail; the rats in which tail colonies did not grow survived for 7 weeks and were free of tumour at autopsy. Injection of the cells (delayed for 80 min after release of the tourniquet) when the phase of reactive hyperaemia had resolved, increased the incidence of rats in which tail nodules developed. Ten-week old rats were used in this experiment and in the group in which the venous outflow was arrested for 5 s to " hold " temporarily the cells in the tail, all rats developed tail tumour nodules; these were more numerous than in rats in which the blood flow remained intact, and the rats also died earlier.

*Local irradiation of tail.*—Local irradiation of the tail with a single dose of

TABLE I

| | Treatment | Day of death (no. of rats)* | No. of rats with tail nodules | No. of rats with tumour destroying lumbosacral muscle and bone | No. of rats with paraplegia at death |
|---|---|---|---|---|---|
| I | Nil | 14(7), 16(1) | 0 | 8 | 8 |
| II | 750 rad LTI | 15(7), 27(1) | 1 | 8 | 8 |
| III | Cells " held " in tail for 5 s; 750 rad LTI | 15(3), 17(2) 19(1), 20(2) | 6 | 8 | 5 |
| IV | Cells " held " in tail for 30 s; 750 rad LTI | 15(1), 16(1), 17(1), 20(2), 22(1), 24(1), 34(1) | 5 | 8 | 6 |
| V | Cells " held " in tail for 30 s; 750 rad LTI, 750 rad to tail | 15(1), 16(1), 17(1), 20(2), 21(1), 52(1), 64(1) | 1 | 8 | 6 |

* Rats with paraplegia, large abdominopelvic masses of tumour or gross dyspnoea and anaemia were killed and scored as deaths on the same day.

Five groups of 6-week old female rats (8 rats per group) injected intravenously with $5 \times 10^4$ W-256 cells (0·1 ml) into distal third of a lateral tail vein. In Groups III, IV and V the venous outflow from the tail was occluded by digital compression while the cells were being injected and for 5–30 s after the injection was completed to " hold " cells in the vasculature of the tail. Rats in Groups II to V were given 750 rad to the thorax approximately 1 h later, and in Group V the tail also was irradiated locally (1 h after injection) with 750 rad.

TABLE II.—*The Influence of rate of Flow of Blood in the Tail on Local Trapping and Growth of Tumour Cells in the Tail, and on Survival of Rats Measured 7 Weeks after Injection of Cells*

| | Treatment | Incidence of tail colonies (day colonies become palpable) | Survival (day of death)* |
|---|---|---|---|
| I | No occlusion | 4/4 (19, 19, 23, 34) | 0/4 (19, 34, 34, 52) |
| II | Arterial occlusion for 15 min; cells injected 20 s later when tail showed reactive hyperaemia | 1/4 (34) | 3/4 (41) |
| III | Arterial occlusion for 15 min; cells injected 80 min after occlusion when hyperaemia had disappeared | 3/4 (11, 19, 26) | 1/4 (23, 41, 45) |
| IV | Cells injected immediately after pressure was applied to the tail to obstruct venous outflow, and maintained for 5 s after the injection | 4/4 (11, 16, 19, 19) | 0/4 (11, 19, 23, 31) |

* Deaths were due to primary growth of injected tumour cells in lungs, and in bone and muscle of the lumbo-sacral regions, as well as to secondary seeding in the lungs from primary growth in the spine, pelvis and tail.

Four groups of 10-week old rats (4 rats per group) were injected intravenously with $10^4$ W-256 cells suspended in 0·1 ml into the distal third of the tail under anaesthesia; the entire tail of each rat was then enclosed in a lead tube (4 mm thick wall) and the remaining unshielded parts of the whole body irradiated with 500 rad.

2000 rad x-rays did not affect the incidence of tail nodules in rats injected intravenously with $10^5$ W-256 cells, irrespective of whether the tails were irradiated 15 min or 10 days before injection of the cells (results not tabulated).

*Growth of subcutaneously injected W-256 cells in tail*

Tumour nodules developed much more slowly in the tail when W-256 cells were injected subcutaneously instead of intravenously. Three weeks after subcutaneous injection of 6 rats with $10^3$ W-256 cells in 0·1 ml into the middle third of the tail no nodules were palpable; by the fourth week 2 rats had formed single small (<2 mm diameter) nodules at the site of injection, and after 8 weeks a nodule had grown in all rats. Tail nodules produced by an intravenous injection were palpable within 3 weeks in over 50% of rats and were invariably multiple (Fig. 2), and it is assumed that each nodule is produced by the growth of one, or possibly a few, cells. When the number of W-256 cells injected subcutaneously into the tail was increased to $10^4$ or $10^5$ cells, palpable tumour developed in all rats within 2–3 weeks.

*Effect of heparinization of rats on venous trapping of tumour cells*

The incidence of tail tumour nodules and of growth of tumour cells trapped in the lumbosacral regions was only slightly reduced in rats injected with heparin intraperitoneally before or after the intravenous injection of tumour cells, and which were given LTI so as to sterilize the tumour cells which reached the lungs (Table III). Treatment with the anticoagulant did appear to increase survival, measured at 6 weeks, particularly if early deaths due to haemorrhage caused by heparin are excluded. The results obtained indicate that in heparinized rats fewer intravenously injected tumour cells failed to reach the lungs and were trapped *en route*. Nevertheless, despite the intensive anticoagulant treatment used in these experiments, trapping and growth of tumour cells *en route* to the lungs did occur in over 50% of rats. Calculations based on previous data showed that after intravenous injection of heparinized rats with W-256 cells about 0·001% of the injected cells remained trapped in the hindquarters of rats 4 h after injection (van den Brenk, 1973*c*). This proportion corresponds to approximately one cell in $10^5$ intra-

TABLE III.—*Effect of Heparinization on Growth of Intravenously Injected Tumour Cells Trapped* en route *to the Lungs*

| | Treatment | No. of rats with tail nodules (day of appearance) | No. of rats with lumbosacral growth and/or tail nodules | Survivors† at 6 weeks |
|---|---|---|---|---|
| A | | | | |
| I | No heparin | 8 (9, 9, 12, 12, 12, 15, 16, 16) | 8 | 4 |
| II | 250 i.u. heparin 10 min before and 4 h after cells (intraperitoneal injections) | 3* (12, 12, 16) | 4* | 1* |
| III | 250 i.u. heparin 24 h after cells (intraperitoneal injections) | 8 (12, 12, 12, 12, 12, 12, 12, 15) | 8 | 5 |
| B | | | | |
| I | No heparin | 6 (12, 20, 23, 27, 36, 36) | 8 | 4 |
| II | 250 i.u. heparin added to intravenously injected tumour cell suspension | 3 (16, 23, 34) | 5 | 8 |
| III | 250 i.u. heparin injected intraperitoneally 10 min before tumour cells | 7 (16, 16, 20, 23, 27, 29, 34) | 7 | 5 |

* Four rats in this group died within 24 h of injection of tumour cells from intraperitoneal haemorrhage.

† Paraplegic and moribund rats were killed and scored as deaths.

(A) 5-week old female rats injected intravenously with $10^5$ W-256 tumour cells into a tail vein; in all rats venous compression was applied to base of tail immediately before and for 10 s after the injection of cells, and 2 doses of 750 rad LTI given 2 and 5 days respectively after the tumour cell injections; (B) 10-week old female rats injected intravenously with $10^5$ W-256 tumour cells (no vascular compression) and given 1000 rad LTI 24 h after the injection. (Each of the 3 subgroups in A and B comprised 8 rats.)

venously injected W-256 cells. The $ED_{50}$ value in rats for W-256 cells injected into muscle is <10 cells (van den Brenk *et al.*, 1973*a*). The incidence of growth of tumour due to trapping *en route* to the lungs obtained in the present series of experiments (Tables I–III) indicates that the probability of survival and clonogenic growth of intravascularly trapped tumour cells is at least as great as that of intramuscularly or subcutaneously injected cells.

*Studies of trapping using intravenously injected labelled W-256 cells*

*Dynamics of trapping in the lungs.*—A trace of the signal recorded over the lungs of a rat injected intravenously with $10^6$ W-256 cells labelled with $^{125}$IUDR *in vitro* is shown in Fig. 3. The labelled cells were washed thoroughly before being injected to remove unincorporated nuclide. The trace shows that it took several min before the majority of injected cells had entered the lungs and that practically no cells disappeared from the lungs for at least 90 min after injection. However, in preparing labelled cells for injection of rats, the Nigrosin exclusion test showed that after repeated washing to remove unincorporated label, 5–20% of the cells were often stained (dead). Rapid losses from the lungs of the latter and of any unincorporated $^{125}$IUDR injected together with the cells probably accounted for early rapid decreases in signal obtained in most experiments within the first 30 min after injection (Fig. 4), in contrast to the result shown in Fig. 3. Allowing for this artefact, it is seen that W-256 cells trapped in the lungs of untreated rats were destroyed more rapidly (particularly during the first 3–4 h after injection) than in rats pre-treated before injection with heterologous rabbit anti-rat lymphocytic serum (ALS) or with LTI. These observations are in agreement with the corresponding data obtained for tumour colony forming efficiency (CFE) in the lungs reported on previously (van den Brenk *et al.*, 1973*b*, 1974*b*). Figure 4 also shows that treatment with LTI of rats which had been previously immunized against allogeneic W-256 tumour cells also decreased the rate of tumour cell destruction during the first 3–4 h after injection, but thereafter the signal de-

FIG. 3.—Radioactive signal over the lungs of an anaesthetized rat produced by intravenous injection (IVI) of $10^6$ W-256 cells labelled *in vitro* with $^{125}$IUDR; the trace (read from right to left) shows that tumour cells continue to accumulate in the lungs for about 5 min after IVI and that no loss of trapped cells occurred for $1\frac{1}{2}$ h after IVI.

creased more rapidly, which is considered to reflect the destruction of tumour cells precipitated by the onset and manifestation of the immune reaction. In untreated mature rats, most intravenously injected tumour cells had disappeared from the lungs and been destroyed 24 h after injection; this is shown by the excretion of labelled iodine in the stomach and urine and by its incorporation in the thyroid (Table IV). Radioassays of lung tissues removed 24 h after W-256 cells (labelled with $^3$H-thymidine *in vitro*) were injected intravenously in 6-week old rats confirmed that LTI enhanced survival of the tumour cells in the lungs (Table V).

*Effects of venous stasis on trapping* en route *to the lungs*.—Occlusion of the venous outflow from the tail while $^{125}$IUDR labelled W-256 cells were being injected intravenously into a tail vein prevented further flow of tumour cells to the lungs (Fig. 5). When the tourniquet was released to restore the flow of blood in the tail, the flow of labelled tumour cells to the lungs was very sluggish in normal rats, whereas restoration of the blood flow produced a rapid clearance of cells from the tail to the lungs in heparinized rats. In both untreated and heparin treated rats, massaging the tail after release of the tourniquet

FIG. 4.—Radioactive signals over the lungs obtained after the intravenous injection of $10^6$ W-256 cells labelled with $^{125}$IUDR *in vitro* in 4 anaesthetized 10-week old rats which had been treated as follows: (a) no previous treatment (●——●); (b) 1000 rad LTI given 7 days before i.v. injection of labelled cells (○——○); (c) intramuscular injection of $10^3$ W-256 cells (not labelled) in right leg (to grow the tumour and immunize the rat) and 1000 rad LTI, both given 15 days before i.v. injection of labelled cells (△——△); (d) 0·5 ml ALS given 48 h before and repeated 2 h before i.v. injection of labelled cells (□——□). The traces have been normalized by plotting the early post-injection peak value for the radioactive signal in each rat as 10 units.

increased the rates of flow of cells to the lungs, which demonstrated that venous stasis had caused trapping of tumour cells in the vasculature of the tail. An intraveous injection of 1 ml isotonic saline into the distal third of the tail also mobilized a proportion of trapped tumour cells. The various changes did not differ when the tail of the rat had been irradiated locally with a single dose of 1500 rad 7 days before the intravenous injection.

*Distribution of tumour cells trapped in tails of rats.*—Radioassays were performed of serial segments (1 cm long) of the tails of rats which were amputated 45 min after the W-256 cells, labelled with $^{125}$IUDR *in vitro*, were injected intravenously; venous outflow was occluded for 5 s after the injection was commenced. The results obtained showed that the concentration of trapped cells was highest at the site of injection and decreased rapidly towards the base of the tail (Fig. 6). Tail colonies were generally most numerous in the proximal parts of the tail (Fig. 2). This difference between trapping and colony distribution appears to be due partly to thickness of tail

TABLE IV.—*Assays of $^{125}I$ Present in Organs Removed from a 6-Week Old Female Rat, 24 h after Intravenous injection of* $5 \times 10^6$ *W-256 Cells which had been Labelled* in vitro *with $^{125}IUDR$*

| | Activity (ct/s/g tissue) |
|---|---|
| Lung | 30·3 |
| Heart | 0·6 |
| Thymus | 0·8 |
| Liver | 1·0 |
| Kidney | 0·8 |
| Spleen | 1·8 |
| Stomach (wall) | 5·6 |
| Stomach (contents) | 17·0 |
| Bladder (wall) | 10·0 |
| Urine | 6·9 |
| Submandibular salivary gland | 0·8 |
| Thyroid | 1806·0 |

TABLE V.—*Tritium Activities in Lungs and Thymus, 24 h after* $5 \times 10^6$ *W-256 Cells Labelled* in vitro *with $^3H$-thymidine* (Groups A and B) or 50 $\mu Ci$ $^3H$-thymidine (Group C) were Injected Intravenously in 6-Week Old Rats*

| Group (no. of rats) | $^3H$-activity (net ct/s) of whole organ (mean ± s.e.) Lungs (I) | Thymus (II) | Ratio (I/II) |
|---|---|---|---|
| A (5) | 820±120 | 30±6 | 27 |
| B (5) | 180±80 | 14±1 | 13 |
| C (3) | 2260±400 | 1060±140 | 2 |

* $2 \times 10^6$ W-256 cells per ml Medium 199 (containing 10% horse serum v/v); 10 $\mu$Ci thymidine-6-$^3H$ (specific activity 27 Ci/mmol) per ml final concentration was added to the cell suspension which was gassed (95% $O_2$/5% $CO_2$) and incubated for 1 h at 37°C; the cells were washed 3 times with ice-cold Tyrode solution before injection.

Rats in Group A only were given 1000 rad LTI 7 days before the injection of tumour cells.

increasing from tip to base, but colony formation may also depend on relative vascularity and heat loss, which would cause tissue temperature and metabolic rate to increase towards the base of the tail.

*Assay of injected tumour cells recovered from the blood of rats.*—Arterial blood recovered from rats exsanguinated *via* the abdominal aorta during intravenous injection of $10^7$ W-256 cells into a tail vein was assayed for the presence of tumour cells by intravenously injecting the blood in weanling rats, given 570 rad whole body irradiation 24 h before the injection; it failed to produce growth of tumour in the recipients. Similar assays of perfusates collected from the inferior vena cava of rats 30 min or 4 h after rats were injected intravenously with $10^7$ W-256 cells into a tail vein demonstrated the presence of tumour cells. From counts of lung tumour colonies, it was calculated that approximately 0·01 and 0·001% of the intravenously injected cells were recovered 30 min and 4 h after injection of the cells respectively; these results have been described previously (van den Brenk, 1973*c*). It is concluded that a proportion of intravenously injected W-256 tumour cells trap *en route* to the lungs, and that the remainder trap in the pulmonary circulation where very few cells escape and enter the systemic arterial circulation.

### DISCUSSION

Our experiments have shown that a significant proportion of intravenously injected W-256 cancer cells become trapped in the venous system *en route* to the lungs in the rat. Intravascular trapping for injections made into a tail vein occurs principally in venous plexuses in bone and muscle in the lumbosacral region and in the tail. In the lumbosacral region the trapped cells proliferate and form solid tumours which invade muscle and bone and produce paraplegia in a high proportion of animals, and metastasize to the lungs. Tumour colonies associated with the tail veins become palpable in rats which survive for about 9 days or more after the injections. The majority of intravenously injected cells take some minutes to flow from tail to the lungs, and a small but significant proportion may take an hour or longer when no obstruction to the venous flow exists. The rate of flow of cells to the lungs is decreased markedly and tail

FIG. 5.—Effects of the application and release of a venous tourniquet applied to the base of the tail of each rat approximately 1 s after the intravenous injection of 5 × $10^6$ $^{125}$IUDR labelled W-256 cells into the distal third of a lateral tail vein was commenced, on the rates of clearance of tumour cells from the tail vasculature to the lungs: (a) in untreated rats (●——●); (b) in rats given 1000 i.u. heparin intraperitoneally 10 min before injection of tumour cells (○——○); (c) in rats given single dose of 1500 rad x-rays locally to the tail only, 7 days before the injection of cells (▲——▲); (d) in rats given both 1500 rad to the tail (Day −7) and heparin 10 min before IVI (△——△). Tourniquet was released at TR; tail massaged at M and 1 ml isotonic saline was injected intravenously immediately proximal to the site of injection of the tumour at S.

trapping of tumour cells is enhanced markedly by venous stasis induced for a few seconds only immediately after injection; with restoration of flow of blood in the tail, tumour cells slowly leave the tail and may take several hours to enter and trap in the lungs. Despite the restoration of flow of blood in the tail produced by the release of a venous tourniquet, a considerable proportion of the tumour cells leaving the tail *en route* to the lungs enter offshoots and ramifications of the venous system in paravertebral regions and hindquarters, where trapping occurs. This happens in spite of the fact that the main flow of blood to the lungs would seem to be the path of least resistance. We cannot give a satisfactory haemodynamic explanation for this diversion and trapping of cells in the "vertebral venous plexus". It occurs whether venous stasis has been induced or not. It seems possible that under experimental conditions reflex veno-

FIG. 6.—Radioassays of the lungs and of serial 1 cm long segments of the tail of a rat injected intravenously into the tail with $10^6$ $^{125}$IUDR labelled W-256 cells; the venous outflow from the tail was arrested immediately before commencing the injection and for a further 5 s after the injection was completed, and the tail amputated 45 min later.

motor stimulation may cause regional shunt of blood to outlying plexuses. Changes in venous pressure and flow produced by respiratory movements may also affect the shunting of blood to outlying plexuses. It is difficult to define the " main route " of venous flow from the tail to the lungs of the rat based on conventional anatomical descriptions of the anatomy of the venous system of this species (Greene, 1968). Venography showed that the two lateral tail veins used by most experimentalists for intravenously injecting cells and other materials communicate freely by numerous interconnecting channels; the lateral tail veins usually empty into the internal iliac veins but also communicate freely with other pelvic veins, namely obturator, pudendal, haemorrhoidal and gluteal systems (Fig. 1), and with other tributaries including the median sacral (caudal) vein, which enters the junction of common iliac veins, and with the vertebral venous system. Thus, ample opportunity exists for tail vein blood to be shunted into outlying venous plexuses in the lumbosacral and gluteal regions.

The phenomenon of venous trapping of tumour cells is cogent to the lung colony assay technique for measuring capacity for clonogenic growth of single tumour cells *in vivo*; more importantly, it appears to have a bearing on the haematogenous spread of cancer cells in the body. It is asserted that although most metastases are produced by multicellular tumour emboli, most extrapulmonary metastases (other than those produced by tertiary dissemination from established lung metastases) are presumably attributed to the failure of the pulmonary capillaries to arrest single tumour cells, which are then distributed to the various organs *via* the systemic arterial circulation (Willis, 1948). However, convincing clinical evidence provided by Batson (1940) and supported by Franks (1953, 1956) showed that metastases in bone from carcinoma of the prostate result principally from venous trapping. The experimental studies described in this paper provide direct proof that under experimental conditions this route of dissemination does occur and can be a major cause of death of rats under circumstances in which the vast majority of injected blood-borne tumour cells trap in the lungs. Both experimental and clinical findings indicate that the lungs, where most blood-borne tumour cells trap, provide relatively adverse conditions for tumour cell growth. Thus, in the intact lungs of adult rats and mice intravenously injected allogeneic and syngeneic cancer cells trap but grow poorly, since CFE is low (0·01% or less), in contrast to higher values for CFE obtained in the unirradiated young (weanling) rat or in the adult when its lungs have suffered damage by x-radiation, when CFE increases 10- to 100-fold (van den Brenk *et al.*, 1973*b*; Withers and Milas, 1973; van den Brenk and Kelly, 1974*c*). Furthermore, it has been shown that CFE in the lungs is also very low for multicellular aggregates of spontaneous murine mammary carcinomata injected intravenously in unirradiated syngeneic recipient mice, *i.e.* for tumour emboli which *cannot fail to trap in the lungs*, and that CFE is similarly increased after local irradiation of the lungs in this system (Thompson, 1974). In human cancer many free cancer cells are frequently present in the venous blood, *en route* to the lungs, but fail to produce lung metastases (Willis, 1948; Cole *et al.*, 1961). It follows that although the lungs have the priority of trapping blood-borne single cancer cells (or aggregates) the yield of pulmonary metastases is often lower than expected, and the yields of blood-borne metastases produced by a variety of commoner forms of cancer (including breast and urogenital tract) may be as high or higher in other organs. This suggests that a major physiological mechanism resides in the lungs, which adversely affects the survival of cancer and probably other foreign cells, and that this mechanism differs in its stimulation, rate of onset and other characteristics from those of host *versus* tumour immunity.

Reports that anticoagulant therapy reduced the incidence and growth of metastases from tumours transplanted in animals (Wood *et al.*, 1961) and the incidence of cancer in humans (Michaels, 1964) have evoked considerable interest. The rationale for anticoagulant and fibrinolytic treatments seems to be based largely on the concept that deposition of fibrin facilitates intravascular arrest and attachment of blood-borne cancer cells to endothelial surfaces, and thereby promotes tumour cell survival and its growth. Since lung endothelium is rich in plasminogen activator enzymes, which would increase fibrinolysis, this might act to the detriment of the trapped cancer cells; a defect in the blood-clotting mechanism (fibrinogen production) might act likewise. However, CFE of W-256 cells in the lungs was not affected by treatment of rats with heparin (van den Brenk *et al.*, 1974*b*), but treatment with heparin did slightly increase the survival of rats injected intravenously with W-256 tumour cells, by reducing the degree of trapping of injected cells *en route* to the lungs. Consequently, we suggest that beneficial effects of anticoagulant therapy on metastasis may be due largely to mobilization of cancer cells in the blood and increased trapping of the cells in the lungs, where biochemical conditions are less favourable for the support of tumour cell survival and growth than in certain other organs. Clonogenic growth of W-256 cells trapped in the vasculature of muscle and bone seems to occur more efficiently than in lungs. It has been reported that growth of W-256 tumour in bone appears to be associated with osteolytic actions of the tumour cell (Raue *et al.*, 1972; Powles *et al.*, 1973).

CFE of W-256 and Y-P388 tumour cells in the lungs is much higher in 3-week old (weanling) rats than in older rats; this decrease in CFE occurs very rapidly during the third week of postnatal life (van den Brenk *et al.*, 1973*a*). We suggest that maturation of pulmonary tissues which occurs during postnatal development markedly enhances the resistance of the lungs to the survival and growth of cancer cells, and possibly other foreign cells which become trapped in the organ; the *milieu interieur* in the adult organ rapidly loses its growth supporting qualities, which results in the increased rejection and death of "grafted" cells. This protective physiological function of lung tissue against "hostile" elements in the circulation brings to mind a somewhat similar rapid inactivation of certain vasoactive hormones (*e.g.* bradykinin) brought by venous blood to the organ (Vane, 1969).

Rejection of cancer cells by the lungs is decreased markedly by local irradiation (van den Brenk *et al.*, 1973*a*) and by non-specific inflammatory reactions induced in the lungs by a variety of agents (van den Brenk *et al.*, 1974*b*), and is counteracted by anti-inflammatory agents (van den Brenk *et al.*, 1974*a*). Anti-inflammatory agents also decrease osteolytic destruction of bone by tumours (Powles *et al.*, 1973). By analogy, it follows that prevention of lung damage and conservation of pulmonary functions would seem to warrant serious consideration with respect to spread of cancer in man and its treatment.

### REFERENCES

BATSON, O. V. (1940) Function of Vertebral Veins and Their Roles in Spread of Metastases. *Ann. Surg.*, **112**, 138.

BURCH, W. M. (1972) A Study of $^{32}$P Uptake by Tumours *in vivo* Based on Measurements of Bremsstrahlung and Čerenkov Emission. *Ph.D. Thesis, University of London.*

COLE, W. H., McDONALD, G. O., ROBERTS, S. S. & SOUTHWICK, H. W. (1961) *Dissemination of Cancer. Prevention and Therapy.* New York: Appleton-Century-Crofts.

FRANKS, L. M. (1953) The Spread of Prostatic Carcinoma to the Bones. *J. Path. Bact.*, **66**, 91.

FRANKS, L. M. (1956) The Spread of Prostatic Cancer. *J. Path. Bact.*, **72**, 603.

GREENE, E. C. (1968) *The Anatomy of the Rat.* New York: Hafner Publishing Co.

MICHAELS, L. (1964) Cancer Incidence and Mortality in Patients Having Anticoagulant Therapy. *Lancet*, ii, 832.

POWLES, T. J., CLARK, S. A., EASTY, D. M., EASTY, G. C. & MUNRO NEVILLE, A. (1973) The Inhibition by Aspirin and Indomethacin of Osteolytic Tumour Deposits and Hypercalcaemia in Rats with Walker Tumours, and Its Possible Application to Human Breast Cancer. *Br. J. Cancer*, **28**, 316.

RAUE, F., MINNE, H., BELLWINKEL, S. & ZEIGLER, R. (1972) Studies on the Hypercalcaemic Syndrome in Rats with Walker Carcinosarcoma 256. *Acta endocr., Copenhagen*, Suppl., **159**, 71.

THOMPSON, S. C. (1974) Pulmonary Tissue Changes and Their Effect on Metastatic Growth of Mouse Tumours. *Ph.D. Thesis, University of London.*

VAN DEN BRENK, H. A. S., SHARPINGTON, C. & ORTON, C. (1973*a*) Macrocolony Assays in the Rat of Allogeneic Y-P388 and W-256 Tumour Cells Injected Intravenously: Dependence of Colony Forming Efficiency on Age of Host and Immunity. *Br. J. Cancer*, **27**, 134.

VAN DEN BRENK, H. A. S., BURCH, W. M., ORTON, C. & SHARPINGTON, C. (1973*b*) Stimulation of Clonogenic Growth of Tumour Cells and Metastases in the Lungs by Local X-radiation. *Br. J. Cancer*, **27**, 291.

VAN DEN BRENK, H. A. S. (1973*c*) Measurements of Tumour-cell Radiosensitivity *in vivo* Using Lung Macrocolony Assay: Dose-Survival Artefact Due to "Tail Trapping". *Int. J. radiat. Biol.*, **23**, 631.

VAN DEN BRENK, H. A. S., KELLY, H. & ORTON, C. (1974*a*) Reduction by Anti-inflammatory Corticosteroids of Clonogenic Growth of Allogeneic Tumour Cells in Normal and Irradiated Tissues of the Rat. *Br. J. Cancer*, **29**, 365.

VAN DEN BRENK, H. A. S., STONE, M., KELLY, H., ORTON, C. & SHARPINGTON, C. (1974*b*) Promotion of Growth of Tumour Cells in Acutely Inflamed Tissues. *Br. J. Cancer*, **30**, 246.

VAN DEN BRENK, H. A. S. & KELLY, H. (1974*c*) Potentiating Effect of Prior Local Irradiation of the Lungs on Pulmonary Metastases. *Br. J. Radiol.*, **47**, 332.

VANE, J. R. (1969) The Release and Fate of Vaso-active Hormones in the Circulation. *Br. J. Pharmac.*, **35**, 209.

WILLIS, R. A. (1948) *Pathology of Tumours.* London: Butterworth & Co. Ltd.

WITHERS, H. R. & MILAS, L. (1973) Influence of Pre-irradiation of Lung on Development of Artificial Pulmonary Metastases of Fibrosarcoma in Mice. *Cancer Res.*, **33**, 1931.

WOOD, S. JR, HOLYOKE, E. D. & YARDLEY, J. H. (1961) Mechanisms of Metastasis Produced by Blood-Borne Cancer Cells. *Can. Cancer Conf.*, **4**. London: Academic Press.